# Association of diet with intestinal permeability and inflammation markers in patients with type 1 diabetes

**DOI:** 10.3892/br.2026.2181

**Published:** 2026-07-29

**Authors:** Sofija Ivanova, Aleksejs Fedulovs, Kaspars Jēkabsons, Inese Siksna, Una Riekstiņa, Leonora Pahirko, Jeļizaveta Sokolovska

**Affiliations:** 1 Faculty of Medicine and Life Sciences, University of Latvia, LV-1004 Riga, Latvia; 2 Risk Assessment and Epidemiology Unit, Institute of Food Safety, Animal Health and Environment ‘BIOR’, LV-1076 Riga, Latvia; 3 Faculty of Science and Technology, University of Latvia, LV-1004 Riga, Latvia

**Keywords:** diet, gut permeability, intestinal permeability, lipopolysaccharide, lipopolysaccharide-binding protein, calprotectin, endotoxemia, inflammation, type 1 diabetes

## Abstract

Nutrition can affect gut dysbiosis and low-grade inflammation in type 1 diabetes (T1D). The present cross-sectional study aimed to explore the association between dietary factors and inflammatory and endotoxemia markers in patients with T1D. Participants completed a 24-h dietary recall, a 3-day food record and a food frequency questionnaire, and serum levels of inflammatory and endotoxemia markers, and fecal calprotectin levels were measured. In the T1D group, higher high-sensitivity C-reactive protein levels were associated with a higher intake of hot drinks (such as coffee, tea and cocoa) [OR, 3.95 (95% CI, 1.41-11.08), P=0.009] and legumes [OR, 4.91 (95% CI,1.38-17.41), P=0.014], and a lower intake of protein [OR, 0.27 (95% CI, 0.08-0.87), P=0.028], fat [OR, 0.29 (95% CI, 0.09-0.93), P=0.037], energy [OR, 0.26 (95% CI, 0.09-0.77), P=0.015] and fiber [OR, 0.34 (95% CI, 0.12-0.99), P=0.047]. Higher lipopolysaccharide (LPS) levels [OR, 2.07 (95% CI, 1.17-8.02), P=0.022]) and LPS/high-density lipoprotein ratio [OR, 2.71 (95% CI, 1.06-6.95), P=0.038] were associated with an increased consumption of red meat. In addition, fecal calprotectin levels were positively associated with higher protein consumption [OR, 3.37 (95% CI, 1.14-9.95), P=0.028]. In conclusion, the present exploratory study identified several associations between dietary choices and markers of intestinal permeability and inflammation in T1D, highlighting the need for further intervention studies investigating intestinal permeability.

## Introduction

Type 1 diabetes (T1D) is an autoimmune disease characterized by absolute insulin deficiency and dependence on insulin injections. In poorly controlled T1D, quality of life can notably deteriorate, mainly due to complications of T1D, including retinopathy, nephropathy, neuropathy, and cardiovascular and neurocognitive diseases (1,2). T1D is characterized by chronic systemic low-grade inflammation, which usually develops gradually without specific symptoms, but can contribute to the progression of diabetic complications (3). Endotoxemia, or the presence of lipopolysaccharide (LPS), an outer membrane component of gram-negative bacteria, in the bloodstream is one of the possible contributors to this low-grade inflammation. Endotoxemia is linked to an increased risk of systemic cardiometabolic disorders, visceral fat accumulation and the progression of diabetic complications (4). It can result from bacteria colonizing various body regions, including the oral cavity, gastrointestinal tract, genitourinary system and respiratory pathways (5). Impaired integrity of the intestinal wall, or increased gut permeability, is one of the major sources of endotoxemia (6). Therefore, LPS activity, LPS/high-density lipoprotein (HDL) ratio, and the concentration of LPS-binding protein (LBP), and endogenous anti-endotoxin core antibody (EndoCAb) IgG and IgM in the circulation are considered to be functional markers of intestinal integrity and permeability (4,7). Intestinal inflammation can also contribute to systemic inflammation. Fecal calprotectin, an inflammatory marker, is higher in individuals with T1D and diabetic nephropathy, as compared with in patients with normal kidney function, further confirming the role of intestinal health in the progression of complications of diabetes (8).

It is known that diet affects the health of the gastrointestinal tract via changes in intestinal integrity and the composition of the gut microbiota (3,9,10). Therefore, nutrition may serve a role as a disease-modifying factor in T1D through its anti-inflammatory and pro-inflammatory properties (1,3). The main nutrients that positively affect the microbiota and intestinal health are fiber, probiotic bacteria, polyphenols, flavonoids and omega-3 fatty acids (11-13). On the other hand, saturated fatty acids, simple carbohydrates, artificial sweeteners and a lack of fiber are dietary factors that may cause dysbiosis (11,14,15). A healthy diet, consistent with dietary recommendations, may be considered anti-inflammatory because it provides all of the aforementioned nutrients (16). Patients with T1D do not have specific nutritional recommendations according to the International Diabetes Federation (17); therefore, the main nutritional recommendations for patients with T1D are a seasonal diet, including as much local products as possible, with a reduced consumption of processed products, salt, sugar and saturated fat (16).

To the best of our knowledge, there are limited available publications observing the association between nutrition and endotoxemia and inflammatory markers in patients with T1D. Therefore, the present cross-sectional study aimed to explore the association between dietary factors and serum levels of inflammatory [high-sensitivity C-reactive protein (hs-CRP)] and intestinal integrity markers (LPS, LPS/HDL ratio, LBP, EndoCAb IgG and IgM), and fecal calprotectin levels in patients with T1D.

## Materials and methods

### Study design and participants

The present quantitative cross-sectional study is part of the longitudinal LatDiane study, which commenced in 2013 (18,19). Recruitment of participants took place between January 15 and August 31, 2021, at the Clinical Research Center of the University of Latvia (Riga, Latvia). Details about the present study were shared via the website and social media channels of the University of Latvia. The protocols of the general LatDiane study, and the present sub-study devoted to nutrition, inflammation and gut health, were approved by the Latvian Central Ethics Committee [approval no. 01-29.1/3 (July 10, 2013)] and its further amendments (clearance no. 1/19-10-01, issued on October 1, 2019; no. 01-29.1/2226, dated April 14, 2020). Written informed consent was obtained from all subjects.

The individuals were split into two research groups: Patients with T1D (n=74) and a control group (n=33), which consisted of otherwise healthy individuals with normal glucose metabolism according to the American Diabetes Association criteria (20). The exclusion criteria for both groups were inflammatory bowel disease (Crohn's disease or ulcerative colitis), celiac disease, pregnancy, acute gastrointestinal infection within 2 months of the planned fecal collection, clinical signs of acute inflammation and fever. Demographic data (age and sex), anthropometric data (weight, height, hip and waist circumference) and nutritional data were collected. In addition, blood serum and fecal samples were obtained for the analysis of inflammatory and endotoxemia markers. Information about the history of medication use and self-reported gastrointestinal symptoms was also collected.

### Collection of nutritional data

Three methods were used to obtain information about the dietary habits of the participants: A food frequency questionnaire (FFQ), a 24-h recall and a 3-day dietary record.

Data about participant intake of energy, macronutrients and fiber were obtained from the 24-h recall and 3-day dietary record. Participants were asked to fill in the food diaries during 2 workdays and 1 weekend day. For accurate data assessment, participants were asked to use a validated portion size picture book or food weighing. The average amount of macronutrients and energy for 4 days (from 24-h recall and 3-day dietary records together) was calculated. Considering that the recommended amount of energy, as well as protein, carbohydrate and fat, is different for women and men, the obtained data on the nutrient intake of the participants were analyzed by sex. The nutritional data and participants' eating habits were compared with the adult nutritional recommendations provided by the Latvian Ministry of Health, which are based on the World Health Organisation’s and Nordic Nutrition recommendations (16,21). Specifically, collected data were compared with the recommended daily energy and macronutrient amounts for adults in the age group 31-60 years, as the median study participant age was 44 (34.93-52.0) years in the T1D group and 37 (32.17-45.79) years in the control group.

The FFQ included 118 different food items (in standard portions) and participants were asked to mark the frequency of their consumption during the past 12 months by choosing one of 10 answer options: ‘Don't use’, ‘A few times a year (1-4 times)’, ‘Once a month’, ‘2-3 times a month’, ‘Once a week’, ‘2-3 times a week’, ‘4-6 times a week’, ‘Once a day’, ‘2-3 times a day’ or ‘4 times a day or more’ (Data S1). For more convenient data analysis, all products were grouped into 27 food groups (Table SI). In the subsequent data analysis, an ‘annual consumption coefficient’ was used, calculated based on the obtained FFQ data. For example, if a product was consumed ‘once a day’, a coefficient of 365 was assigned; if the answer was ‘once a month’, 12 was used, and so on. Subsequently, comparing these responses with dietary recommendations, all answers were converted to times per week, assuming there are 52 weeks in a year.

The dietary data analysis was conducted by the Institute of Food Safety, Animal Health, and Environment ‘BIOR’ (Riga, Latvia) using a custom program based on the Microsoft Dynamics AX 2009 platform (Microsoft Corporation). This program utilizes the Latvian Food Composition Database, which was developed to analyze food consumption data from the ‘Food Consumption Study of the Latvian Population (2012-2013)’ (22) and continues to provide data for other studies on food consumption within the Latvian population. The program includes algorithms and software designed to automate the calculation of daily nutrient intake and/or food group consumption using the national food consumption database, with results presented as average intake values (22).

Data from the FFQ regarding consumption of the 27 aforementioned product groups were categorized as either ‘low consumption’ or ‘moderate-to-high consumption’ based on the median consumption values derived from the present study participants' FFQ data (Table SII). The categorized food consumption data were analyzed for both study groups, the T1D and control groups, in relation to inflammatory and endotoxemia markers.

### Collection of medication history and gastrointestinal symptoms

Information was collected on the medications used by participants, including antihypertensive and cholesterol-lowering drugs, immunosuppressive medications or corticosteroids, as well as medications related to gastrointestinal symptoms, such as acid-reducing agents, antispasmodics and medications used to relieve flatulence, diarrhea or constipation, as well as the use of insulin among participants with T1D (Table I). Participants were asked how often they used each medication (ranging from ‘never’ to ‘every day’). For medications related to gastrointestinal symptoms, participants were divided into two groups: Those who used the medication at least 1-4 times per month or more frequently, and those who used it less than once per month. For the other medications (such as cholesterol-lowering medication), participants were categorized simply as users or non-users.

Information on gastrointestinal symptoms was assessed using a self-reported questionnaire consisting of 18 items that addressed abdominal pain, discomfort and bowel movement disturbances, including diarrhea and constipation. Participants were asked how often and to what extent they experienced these symptoms. The mean value of the gastrointestinal symptom score was then calculated, as previously described (8).

### Collection of laboratory data

Blood samples were collected via venous puncture and were then assayed to assess clinical markers (for example, blood count and clinical chemistry, including HbA1c) in a certified clinical laboratory. For further analysis of endotoxemia markers and hs-CRP, the blood samples were incubated undisturbed for 30 min at room temperature and then centrifuged at 3,220 x g at 4˚C for 15 min. The serum was then removed from the pellet, transferred into 2-ml tubes, frozen and stored at -20˚C until analysis. Biobanking and sample storage were performed in agreement with the procedures of the Genome Database of the Latvian Population (23).

Stool samples were collected within 2 weeks of blood collection. Participants collected their fecal samples at home using the FecesCatcher (Zymo Research Corp.) to avoid contact of the stool with toilet water, urine and disinfectants, and with sterile collection tubes without buffer (the collection date and time were marked). Within 24 h, the samples were delivered to the laboratory for calprotectin measurement in unfrozen samples.

LPS levels in serum were assessed using the Hycult LAL chromogenic endpoint assay (cat. no. HIT302; Hycult Biotech); LBP levels in serum were measured with the Hycult LBP Human ELISA kit (cat. no. HK315-02; Hycult Biotech); and serum EndoCAb IgG and EndoCAb IgM levels were analyzed using Hycult EndoCAb IgG and IgM ELISA kits (cat. nos. HK504-IGG and HK504-IGM; Hycult Biotech). Hs-CRP levels in serum were determined using the Hycult Human hs-CRP kit (cat no. HK369; Hycult Biotech). All assays were performed according to the manufacturer's protocols. Fecal calprotectin was measured with the Alegria^®^ Calprotectin ELISA kit (cat. no. ORG280; Orgentec Diagnostika; Sebia) in a certified clinical laboratory (7).

Serum inflammatory and endotoxemia markers were assessed across three plates. Data were examined for consistency across the plates and only LBP required normalization. The normalization procedure was performed using a location-scale transformation, as described in previous studies (7,24). Fecal calprotectin levels varied in the study group, with only six patients exceeding 50 µg/g and five surpassing 200 µg/g. To minimize variability, values >200 µg/g were treated as outliers for further analysis.

### Data processing and statistical analysis

Data processing and statistical analysis were performed using the statistical software JASP (version 0.18.3.0) (25) and IBM SPSS Statistics (version 22; IBM Corp.). The normality of continuous data was assessed using the Shapiro-Wilk and Kolmogorov-Smirnov tests, supplemented by visual inspection of histograms. Most variables did not meet the assumption of normality; therefore, data are presented as the median and interquartile range (1st and 3rd quartiles). The proportions of categorical variables between the groups were compared using the χ^2^ proportion test. Comparisons between the T1D and control groups were performed using the Mann-Whitney U test. Spearman's rank correlation coefficient was used to assess correlations between two continuous variables. Associations between categorized product consumption (divided by the median into two groups: Low vs. moderate-to-high) and categorized inflammatory and endotoxemia markers (dichotomized by their medians) were assessed using binary logistic regression, with the latter serving as the outcome variable (Table SII). Five models were analyzed in the T1D group and four in the control group, including univariate and multivariate models adjusted for covariates in various combinations. In both studied groups, Model 1 was univariate, Model 2 was adjusted for body mass index (BMI), Model 3 was adjusted for BMI and diabetes duration in the T1D group, and for BMI and glycated hemoglobin (HbA1c) in the control group. In the T1D group, Model 4 was adjusted for BMI, diabetes duration and HbA1c, and Model 5 was also adjusted for gastrointestinal symptom score. In the control group, Model 4 was adjusted for BMI, HbA1c and gastrointestinal symptom score. Multicollinearity in the regression models was assessed using the variance inflation factor. P<0.05 was considered to indicate a statistically significant difference.

## Results

### Characteristics of the participants

The present study comprised two research groups. In the group of patients with T1D (n=74), 38% (n=28) were male and 62% (n=46) were female. In the control group, or participants with normal glucose metabolism (n=33), 42% (n=14) were male and 58% (n=19) were female. The median age of participants in the T1D group was 44.0 (34.9-52.0) years, with a minimum age of 22 years and a maximum of 80 years, whereas the median age in the control group was 37.0 (32.2-45.8) years. Anthropometric parameters did not differ significantly between the studied groups (Table I).

The median duration of diabetes in the T1D group was 21.0 (13.0-31.8) years and some patients had developed complications of diabetes. HbA1c, HDL cholesterol and hs-CRP levels were statistically significantly higher in the T1D group compared with those in the control group (Table I).

### Eating habits

Data from the FFQ were used to analyze the eating habits of the participants during the past 12 months. Study participants consumed cereals and pasta approximately four times a week, potatoes two times a week and bread eight to 10 times a week (at least once daily). Fish and seafood were eaten two to three times a month. Milk and milk products were consumed approximately twice daily by patients with T1D and once daily by the control group. In both studied groups, white and red meat, as well as processed meat and fish products, were consumed two to three times a week, while eggs were consumed approximately three times a week. Patients with T1D consumed vegetable oils and fats, butter and other animal fats equally often (five times a week), whereas the control group consumed more vegetable oils and fats (five times a week) and less butter and other animal fats (once a week). Cooked and fresh vegetables were consumed twice a day in both groups. The participants ate fruits and berries approximately once a day (7.5 times a week in the T1D group and 5.9 times a week in the control group). Legumes, mushrooms (fresh and pickled), pickled and salted vegetables were consumed several times a month, and nuts and seeds were eaten twice a week. The consumption of the mentioned products did not differ significantly in both studied groups (Table II).

Participants drank water ~35.1 times a week, equivalent to approximately five 220-ml glasses of water daily. Analysis of beverage consumption (alcohol, sweetened beverages, water, coffee, tea and cocoa) showed that only sweetened beverage consumption was statistically significantly higher in the control group (P=0.049; Table II), while no significant differences were observed for the other beverage consumptions (all P>0.005; Table II).

Participants ate sweets and/or pastries daily. The control group reported statistically significantly higher consumption of sweets and pastries (P=0.003), while products with sweeteners (artificial and other sugar substitutes) were consumed more frequently by the T1D group (P=0.049) (Table II).

Fast food and snack consumption was recorded on average once a week in the T1D group, and twice a week in the control group. However, the consumption of manufactured sauces, such as mayonnaise and soy sauce, was statistically significantly higher in the control group (P=0.026; Table II).

### Average macronutrient intake

Data from the 24-h recall and 3-day food records were used to analyze the average macronutrient and energy consumption of participants. As shown in Table III, participants in both studied groups had insufficient energy intake; they consumed enough protein [17-20 percentage of total energy intake (E%)], whereas the amount of carbohydrates consumed (32-41 E%) was lower than recommended and fat intake (40-42 E%) was higher than recommended. In addition, fiber intake was insufficient (14.0-23.9 g). The intakes of protein, fat and energy were statistically significantly lower in female patients with T1D compared with in women in the control group (P=0.025, P=0.003, P=0.003, respectively). There were no statistically significant differences between male participants in both studied groups regarding macronutrient and energy consumption.

### Analysis of dietary factors in relation to inflammation and endotoxemia

The levels of inflammatory and endotoxemia markers were compared, according to product consumption patterns, between the high- and low-consumption groups using Mann-Whitney U test (Table SIII). Statistically significant differences were then further analyzed using binary logistic regression with different models to determine the strength and direction of the associations (Tables IV, V, SIV and SV). All analyses were performed separately for the two studied groups.

According to the results of the Mann-Whitney U test, in the T1D group, significantly higher LPS/HDL ratio levels were found in subjects consuming moderate-to-high amounts of cereals and pasta, red meat, and nuts and seeds (P=0.023, P=0.022 and P=0.036, respectively). Patients with T1D consuming higher amounts of red meat and water had higher LPS levels (P=0.046 and P=0.043, respectively), whereas those who consumed more sweets and pastries had lower LPS levels (P=0.023). Patients with T1D and a higher consumption of processed meat and fish had higher fecal calprotectin levels (P=0.017), and those that consumed more legumes had higher hs-CRP levels (P=0.019). Patients with T1D and a higher consumption of sweeteners and products containing them had lower fecal calprotectin and EndoCAb IgM levels (P=0.04 and P=0.008, respectively).

In the control group, subjects consuming more red meat, white meat, vegetables (fresh and cooked), potatoes and sauces had higher LPS levels (P=0.029, P=0.009, P=0.049, P=0.024 and P=0.026, respectively). In addition, the LPS/HDL ratio and LBP levels were higher in those consuming more white meat (P=0.025 and P=0.002, respectively). In the control group, LBP was higher in those consuming more sweetened beverages (P=0.048), and higher hs-CRP levels were observed in control group participants with a higher consumption of mushrooms (pickled and fresh) (P=0.038) and water (P=0.033). EndoCAb IgM levels were higher in those control group participants consuming more water and less hot drinks, such as coffee, tea or cocoa (P=0.015 and P=0.049, respectively). Control group participants with a higher consumption of alcohol (P=0.027), and a lower consumption of milk and milk products, potatoes, butter and other animal fats had higher EndoCAb IgG levels (P=0.009, P=0.043 and P=0.010, respectively).

A total of 18 food groups that demonstrated statistically significant between-group differences in inflammatory markers, based on the Mann-Whitney U test, were further analyzed using binary logistic regression models (Tables IV and SIV).

In the T1D group, higher consumptions of red meat [LPS, Model 2: OR, 2.85 (95% CI, 1.08-7.56), P=0.035; LPS/HDL ratio, Model 2: OR, 2.63 (95% CI, 1.01-6.80), P=0.047] and cereals and pasta [LPS/HDL ratio, Model 2: OR, 2.68 (95% CI, 1.04-6.87), P=0.041] were associated with higher endotoxemia markers (LPS and LPS/HDL ratio) in the models adjusted for BMI. However, after adjusting for diabetes duration and HbA1c, the association became marginally significant (Table IV). Higher consumption of legumes in the T1D group was associated with higher hs-CRP levels in the fully adjusted model 5 [OR, 4.91 (95% CI, 1.38-17.41), P=0.014]. In the T1D group, a higher consumption of sweeteners and products containing them was statistically significantly inversely associated with EndoCAb IgM and calprotectin levels in all applied regression models. In the model 4, adjusted for BMI, Hb1Ac and duration of diabetes, individuals with T1D who reported a higher consumption of sweeteners and products containing them exhibited a 64% reduction in the odds of elevated EndoCAb IgM levels and a 68% reduction in the odds of elevated calprotectin levels (Table IV).

Notably, in the T1D group, the consumption of coffee, tea and cocoa exhibited a pro-inflammatory effect. Higher consumption of these drinks was associated with an approximately four times higher chance of elevated hs-CRP levels (Model 5, P=0.009; Table IV). In the control group, the opposite effect was observed, and a higher consumption of coffee, tea and cocoa was associated with a 92% lower risk odds of high EndoCAb IgM levels (Model 4, P=0.027; Table SIV).

In the control group, a higher consumption of potatoes [Model 4, OR, 0.11 (95% CI, 0.02-0.69), P=0.018] and butter or other animal fats [Model 4, OR, 0.08 (95% CI, 0.08-0.82), P=0.033] in the fully adjusted model was inversely associated with EndoCAb IgG, suggesting an anti-inflammatory tendency (Table SIV). In addition, a higher consumption of manufactured sauces was associated with a five times higher chance of high LPS levels (Model 3, P=0.047, Table SIV). In the control group, a higher consumption of nuts and seeds was associated with six times higher odds of elevated EndoCAb IgM in multivariate logistic regression model 3 adjusted for BMI and HbA1c [OR, 6.65 (95% CI, 1.25-35.37), P=0.026], suggesting a pro-inflammatory tendency (Table SIV).

Analyzing associations between energy, macronutrients and fiber, and inflammatory and endotoxemia markers in the T1D group, higher energy [Model 5: OR, 0.26 (95% CI, 0.09-0.77), P=0.015], protein [Model 5: OR, 0.27 (95% CI, 0.08-0.87), P=0.028) and fat [Model 5: OR, 0.29 (95% CI, 0.09-0.93), P=0.037] intake were associated with lower hs-CRP levels in fully adjusted models (Table V). No statistically significant associations were observed between carbohydrate intake and inflammatory or endotoxemia markers in the T1D group (data not shown).

Higher fiber intake was associated with 66% lower hs-CRP levels when adjusted for BMI and duration of diabetes [Model 4: OR, 0.34 (95% CI, 0.12-0.99), P=0.047), but after adjustment for HbA1c and gastrointestinal symptom score, the significance was lost. Higher protein [Model 5: OR, 3.37 (95% CI, 1.14-9.95), P=0.028] intake was associated with higher fecal calprotectin levels in the T1D group in the fully adjusted model (Table V).

In the control group, higher carbohydrate consumption was associated with 92% lower odds of elevated EndoCAb IgM levels in fully adjusted model 4 [OR, 0.08 (95% CI, 0.01-0.75), P=0.027; Table SV). In the control group, a higher fat intake was associated with higher LPS and calprotectin levels in several multivariate regression models. In addition, higher fiber intake was associated with higher LPS and hs-CRP levels in multivariate models in this group (Table SV).

## Discussion

The present cross-sectional study explored the association of identified dietary factors with intestinal permeability and inflammatory markers in a cohort of subjects with T1D in Latvia. In addition, the nutritional intake of patients with T1D, in relation to standard nutritional recommendations, was reported and compared with generally healthy individuals.

Different associations were observed between dietary components and inflammatory and endotoxemia markers in individuals with T1D and control subjects. This may partially be explained by background differences in the inflammatory and glycemic profiles of the study groups, as well as differences in dietary habits. Notably, patients with T1D had higher levels of hs-CRP and HbA1c than the control group at the time of the sample collection. Patients with T1D also used more antihypertensive and cholesterol-lowering drugs. Differences in gut microbiota, along with genetic and epigenetic factors, may influence nutrient metabolism, intestinal barrier function and immune responsiveness in T1D, contributing to altered inflammatory responses to diet compared with in individuals without diabetes (10).

There are relatively few studies investigating the associations between diet and inflammation or endotoxemia in patients with T1D. Most studies link the effects of diet on endotoxemia and inflammation to gut health and microbiota balance, emphasizing the positive role of fiber and unsaturated fats, and the negative influence of a Western-style diet (4,26,27). The most commonly reported markers of endotoxemia in these studies are LBP and LPS (4). To the best of our knowledge, no previous studies have reported associations between dietary factors and EndoCAb IgG or IgM levels.

FFQ analysis demonstrated that the participants in the current study did not consume enough complex carbohydrates (such as cereals and pasta), vegetables, fruits, fish, legumes, nuts and seeds as recommended by the Latvian national recommendations (16). This explains why the diets were insufficient in fiber (twice less than recommended), increasing the risk of intestinal dysbiosis. Previous studies in the general population have demonstrated that adequate dietary fiber intake has a beneficial effect on intestinal barrier function, reducing LPS and LBP levels (26,28). In addition, a high-fiber diet may help to delay gastric emptying, thus lowering the post-meal glycemic response (29). LBP and inflammatory marker concentrations in plasma are negatively associated with vegetable intake in the general population (28), whereas the data in T1D are controversial (4). The present study demonstrated that higher fiber intake was associated with lower hs-CRP levels in the T1D group in the multivariate regression model. However, in the T1D group, a greater consumption of cereals and pasta was associated with a higher LPS/HDL ratio, whereas a greater intake of legumes was associated with higher hs-CRP levels. Moreover, in the control group, LPS and hs-CRP levels were positively associated with fiber consumption.

The following explanation has been proposed for the observed results. Firstly, considering that participants had adhered to an unbalanced diet for ≥1 year prior to sample collection (as assessed by the FFQ), gut barrier integrity may have been impaired, potentially due to sustained low-fiber and high-fat intake. Additionally, the ‘cereals and pasta’ group includes gluten-containing products. For example, gliadin, one of the proteins in gluten, may increase gut permeability even in individuals without celiac disease (30), potentially leading to endotoxemia and systemic inflammation (31), especially in subjects with T1D who are prone to gastrointestinal disturbances and dysbiosis (8). In addition, an insufficient intake of vegetables, fruits and berries in the diet, as observed in the current study, may markedly decrease antioxidant, polyphenol and vitamin levels, thereby reducing the anti-inflammatory properties of the diet. Moreover, the rare inclusion of fish, nuts and seeds can cause a lack of omega-3 fatty acids, which have anti-inflammatory effects and can improve the intestinal microbiota (11,14,32).

Sweeteners are alternatives to sugar, honey and fructose syrup, which are widely used by subjects with T1D to avoid the consumption of sugar and to minimize postprandial glucose excursions (17,33). Consumption of this product group (‘sweeteners and products containing them’, including artificial sweeteners, stevia and products with them) in the present study was inversely associated with EndoCAb IgM and calprotectin levels in patients with T1D. The impact of sweeteners on gut health, insulin sensitivity and other health outcomes remains controversial due to potential disruptions to gut microbiota and metabolic processes (34-37). Among all sweeteners, stevia is reported as having a range of positive effects, and seems to not notably alter the overall composition or diversity of the gut microbiota (38). In addition, stevia has been shown to exhibit potential anti-inflammatory properties in *in vitro* and *in vivo* studies on mice, reducing LPS-induced inflammation by modulating key inflammatory signaling pathways and increasing anti-inflammatory cytokines (39,40), which is in agreement with the present findings.

In the general population, hot beverages such as coffee, tea and cocoa have been associated with beneficial health effects. Moderate consumption of coffee (less than four cups per day) may exhibit antibacterial properties via phenolic and chlorogenic acids, and anti-inflammatory benefits due to polyphenols and other bioactive compounds (41). However, chronic and excessive caffeine consumption (500-600 mg or more than four cups of coffee per day) may be associated with dysfunction of the gastrointestinal tract, liver, kidneys and skeletal muscles (42). Tea consumption may have a beneficial effect on the health of patients with T1D, largely due to its content of phenolic compounds with antioxidant, anti-inflammatory and immunomodulatory properties, as well as beneficial effects on the composition of the intestinal microbiota (43). Additionally, cocoa-derived procyanidins have been shown to help maintain gut barrier integrity and prevent its disruption, particularly in inflammatory conditions (44). However, despite the beneficial properties of these drinks, they can also impact the absorption of lipids, vitamins, minerals and drugs (45). In the current study, participants consumed a moderate amount of coffee, tea and cocoa, approximately three times per day, and a higher consumption was associated with higher hs-CRP levels in the T1D group. Data from the ATTICA study, conducted in Greece and including 3,042 randomly selected adults, demonstrated positive associations between coffee consumption and several inflammatory markers, including CRP, IL-6, serum amyloid A (SAA) and tumor necrosis factor-α. The authors of this previous study hypothesized that coffee consumption may stimulate IL-6 synthesis, which in turn could enhance hepatic production of CRP and SAA, thereby contributing to a pro-inflammatory profile (46). In another systematic review and meta-analysis of 11 studies, with a total of 61,047 participants, the authors did not find a statistically significant dose-response association between coffee consumption and CRP levels (47). In the Finnish Diabetic Nephropathy Study, which included 1,040 participants with T1D, consumption of more than three cups of coffee per day was associated with a higher risk of metabolic syndrome, and consumption of any amount of coffee was associated with a heightened risk for hypertension (48). Another reason for higher hs-CRP levels in participants with T1D who consume more coffee, tea or cocoa may be the addition of sugar, honey or syrup to these drinks, making them potentially pro-inflammatory, especially when consumed several times a day (49). Additionally, added sugar may negate the beneficial effects of coffee (50), reduce insulin sensitivity and impair glycemic control in T1D (51).

In the current study, participants consumed enough protein, less than the recommended amount of carbohydrates and more than the recommended amount of fat. Lower protein, fat and energy intake was associated with higher hs-CRP levels in the T1D group. This may be a consequence of insufficient nutrition due to the disease burden. However, these macronutrient consumption trends also align with the findings of a dietary study of the Latvian population (22).

The present study observed that higher protein consumption was positively associated with higher fecal calprotectin levels in individuals with T1D. Analyzing the FFQ data, it was concluded that animal protein sources, such as meat, eggs, milk and dairy products, prevailed in the diets of the participants. According to the literature, diets high in animal protein can negatively affect gut health by increasing systemic LPS and CRP, and reducing microbiota diversity, while increasing the levels of potentially harmful trimethylamine N-oxide and IGF-1 (11,12,52). In the present study, red meat consumption in the T1D group was associated with approximately three times higher LPS and LPS/HDL ratio levels, but the statistical significance of the association was lost if the regression model was adjusted for duration of diabetes and HbA1c, indicating that the burden of the disease and the quality of glycemic control serve a major role as predictors of endotoxemia in these patients.

Previous studies have demonstrated that saturated fat consumption is associated with inflammation (53,54), although the impact of fat on intestinal permeability and endotoxemia remains a controversial topic (55,56). The current study did not provide information on the distribution of fat intake (for example, saturated vs. unsaturated fat), but the FFQ data indicated that saturated fats prevailed in the diets of the participants. In turn, vegetable oils, fish, nuts and seeds were consumed less often than recommended in both studied groups. In the control group of the present study, higher LPS levels were associated with higher fat consumption, in alignment with some literature data (26,53,54). However, it was observed that fat intake was inversely associated with hs-CRP levels in individuals with T1D. In addition, in the control group, higher consumption of butter and other animal fats was associated with a 92% lower EndoCAb IgG level. A possible explanation is that a higher dietary fat intake is often accompanied by lower carbohydrate intake, which may reduce glycemic variability, factors that could potentially influence inflammatory responses (57,58).

Regarding milk and dairy products, a systematic review of randomized clinical trials concluded that milk and dairy consumption have no pro-inflammatory effect on healthy adults or overweight/obese adults, with some evidence of anti-inflammatory benefits (59). Dairy products, including butter, contain not only saturated fats but also pentadecanoic acid (15:0), an odd-chain saturated fat linked to a decreased incidence of cardiovascular disease and coronary heart disease events (60). Similarly, conjugated linoleic acid isomers (especially cis-9, trans-11 rumenic acid) from milk and milk products exhibit anti-inflammatory effects (61). Considering that consumption of butter or other animal fats was relatively low in the present study (once a week in the control group and five times a week in the T1D), these findings suggest that a moderate intake of full-fat dairy might have a neutral-to-beneficial effect.

The findings of the present study provide valuable insights into the association between dietary intake, inflammation and endotoxemia in T1D. Some aspects should, however, be considered when interpreting the results. The cross-sectional design does not allow conclusions about causality; therefore, the findings should be viewed as hypothesis-generating for future intervention trials. A relatively large number of statistical tests were performed across several models and outcomes to explore potential associations. Formal multiple-testing corrections were not applied because the primary aim was hypothesis generation, and strict corrections, such as Bonferroni, would be overly conservative and increase the likelihood of type II error in this setting. Instead, the current study focused on the consistency and clinical plausibility of associations across models, including the logistic regression analyses. Nevertheless, the risk of type I error due to the multiple statistical tests performed across the investigated outcomes cannot be excluded. Therefore, the findings should be interpreted as exploratory and confirmed in future studies with predefined hypotheses and appropriate control for multiple statistical testing. Additionally, potential confounding factors, such as physical activity, stress levels, smoking and socioeconomic status, may have influenced the results but were not specifically addressed or analyzed in the present study. Dietary intake was assessed using self-reported methods, which may involve some degree of under- or over-reporting. The control group was relatively small, but as the focus was on individuals with T1D, this does not affect the conclusions of the study. In addition, adherence to diabetes self-management and insulin administration was not assessed, although these factors may influence inflammation and glycemic variability (62). To partly account for this, some of the regression models were adjusted for HbA1c, as an indicator of long-term glycemic control, and for duration of diabetes in the T1D group.

Despite these considerations, the present study has notable strengths. To the best of our knowledge, it is the first study to examine associations between diet and multiple markers of gut permeability and intestinal inflammation in T1D, including fecal calprotectin and endotoxin-binding proteins. Moreover, the use of three complementary dietary assessment methods, 24-h recall, 3-day food diaries and FFQ, enhances the robustness of dietary evaluation.

In conclusion, the present exploratory study identified specific associations between dietary factors and markers of intestinal permeability and inflammation in T1D, highlighting several hypotheses for future intervention studies. Hot beverages, including coffee, tea and cocoa, were associated with pro-inflammatory activity, and future trials should examine dose-response relationships, differences between beverage types and whether effects are modified by glycemic control status. Although sweetener intake was inversely associated with fecal calprotectin and EndoCAb IgM in the present study, the potential impact on gut microbiota and intestinal permeability remains to be clarified. In the control group butter intake was associated with lower EndoCAb IgG levels, suggesting an anti-inflammatory potential that may extend to other dairy products and warrants further investigation, including dose-response relationships. Across all these directions, for future studies, stratification by glycemic control and disease duration (in T1D) is recommended, alongside adjustment for life-style factors as physical activity, stress and sleep quality. To ensure comparability across future studies, it is recommended to include several markers of inflammation and endotoxemia, such as fecal calprotectin, CRP, LBP, LPS, EndoCAb IgG and IgM.

## Supplementary Material

Product groups.

Medians for data grouping.

Levels of inflammatory and endotoxemia markers by frequency of food group consumption in T1D and control groups.

Associations between food group consumption and markers of intestinal permeability and inflammation in the control group.

Associations between macronutrient intake and markers of endotoxemia and inflammation in the control group.

Data S1

## Figures and Tables

**Table I:** Characteristics of the participants.

Variable	Type 1 diabetes group (n=74)	Control group (n=33)	P-value
Male/Female, n (%)	28/46 (38/62)	14/19 (42/58)	0.951
Age, years	44.0 (34.9-52.0)	37.0 (32.2-45.8)	0.022^a^
Height, m	1.7 (1.7-1.8)	1.8 (1.7-1.8)	0.217
Weight, kg	76.6 (67.3-84.0)	76.0 (65.0-84.0)	0.960
BMI, kg/cm^2^	24.9 (22.6-28.4)	24.3 (22.3-27.9)	0.386
Waist circumference, cm	86.0 (75.3-94.0)	84.0 (75.0-89.0)	0.291
Hip circumference, cm	104.0 (97.0-108.0)	101.0 (94.0-105.0)	0.184
Serum glucose, mmol/l	10.3 (7.2-13.4)	5.0 (4.8-5.1)	<0.001^a^
HbA1c, %	7.7 (6.9-9.3)	5.2 (5.0-5.5)	<0.001^a^
Total cholesterol, mmol/l	5.0 (4.5-5.8)	4.7 (4.5-5.1)	0.136
HDL cholesterol, mmol/l	1.5 (1.3-1.9)	1.4 (1.3-1.6)	0.050^a^
LDL cholesterol, mmol/l	3.0 (2.3-3.4)	2.81 (2.4-3.2)	0.769
Triglycerides, mmol/l	1.2 (0.9-1.5)	1.0 (0.8-1.7)	0.205
TSH, mU/l	1.5 (1.1-2.4)	1.3 (0.92-1.9)	0.247
Alanine aminotransaminase, U/l	19.0 (15.0-28.0)	21.0 (17.0-29.0)	0.182
Aspartate aminotransferase, U/l	22.0 (19.0-31.0)	25.0 (22.0-29.0)	0.126
Bilirubin, µmol/l	9.3 (7.0-12.0)	10.5 (7.1-15.0)	0.373
LBP, µg/ml	11.1 (7.9-14.0)	10.1 (7.0-14.2)	0.600
LPS/HDL ratio	0.2 (0.2-0.3)	0.2 (0.2-0.4)	0.269
EndoCAb IgM, MMU/ml	45.8 (33.4-69.5)	48.3 (27.4-69.7)	0.637
EndoCAb IgG, GMU/ml	89.8 (64.2-142.7)	71.1 (55.7-101.5)	0.135
LPS, EU/ml	0.4 (0.3-0.5)	0.4 (0.3-0.5)	0.487
hs-CRP, mg/l	0.8 (0.5-1.8)	0.5 (0.3-0.8)	0.002^a^
Calprotectin, µg/g	5.8 (2.7-16.2)	6.4 (3.9-10.3)	0.585
Diabetes duration, years	21.0 (13.0-31.8)	-	-
Diabetic retinopathy, n (%)	28 (37.8)	-	-
Diabetic nephropathy, n (%)	14 (18.9)	-	-
Arterial hypertension, n (%)	35 (47.3)	6 (18.2)	0.026^a^
CVD, n (%)	9 (12.2)	-	-
Total insulin units, units/kg/day	0.6 (0.5-0.7)	-	-
Use of antihypertensive drugs, n (%)	28 (37.8)	2 (6.1)	<0.001^a^
Use of cholesterol-lowering medications, n (%)	17 (23.0)	2 (6.1)	0.039^a^
Use of immunosuppressive medications or corticosteroids, n (%)	2 (2.7)	1 (3.0)	>0.99
Use of medications related to gastrointestinal symptoms, n (%)	26 (35.1)	10 (30.3)	0.698
Gastrointestinal symptom score	1.0 (0.7-1.2)	0.8 (0.8-1.1)	0.359

^a^P<0.05. For categorical variables, data are presented as counts (%), whereas for continuous variables, data are presented as median (1st and 3rd quartiles). Continuous variables were compared between the groups by the Mann-Whitney U-test, and categorical variable proportions between the groups were compared using the χ^2^ proportion test or Fisher's exact test, as appropriate. Arterial hypertension was classified as systolic blood pressure ≥140 mmHg (18.7 kPa) or diastolic blood pressure ≥90 mmHg (12.0 kPa), or a history of antihypertensive drug treatment. Diabetic retinopathy refers to history of any stage of retinopathy based on medical recordings. CVD was defined as a history of acute myocardial infarction, coronary bypass/percutaneous transluminal coronary angioplasty, stroke, amputation or peripheral vascular disease. Diabetic nephropathy was defined as micro/macro albuminuria or end-stage renal disease. BMI, body mass index; CVD, cardiovascular disease; EndoCAb, endogenous anti-endotoxin core antibody; HbA1c, glycated hemoglobin; HDL, high-density lipoprotein; hs-CRP, high-sensitivity C-reactive protein; LBP, LPS-binding protein; LDL, low-density lipoprotein; LPS, lipopolysaccharide; TSH, thyroid-stimulating hormone.

**Table II:** Summary of food products and beverage consumption among participants.

	Type 1 diabetes group (n=74)		Control group (n=32)		
Food group	Median of average annual ratio	Times/week	Median of average annual ratio	Times/week	P-value
Cereals, pasta	236.8 (129.4-438.3)	4.6	224.3 (113.8-396.9)	4.3	0.921
Potatoes	118.3 (49.5-260.0)	2.3	82.0 (45.1-240.5)	1.6	0.504
Bread	495.0 (280.3-985.6)	9.5	419.5 (166.0-823.8)	8.1	0.386
Vegetables (fresh, cooked)	694.8 (441.2-1243.6)	13.4	689.0 (392.0-1224.5)	13.3	0.765
Pickled and salted vegetables	24.0 (5.0-55.9)	0.5	31.3 (12.0-60.0)	0.6	0.310
Fruits and berries	391.3 (178.4-637.0)	7.5	307.0 (224.6-628.0)	5.9	0.828
Legumes	30.0 (12.0-52.0)	0.6	52.0 (16.5-52.0)	1.0	0.318
Nuts and seeds	100.3 (29.0-198.5)	1.9	92.3 (40.5-202.0)	1.8	0.393
Mushrooms (fresh and pickled)	24.0 (5.0-42.0)	0.5	28.3 (5.0-42.0)	0.5	0.806
Red meat	106.3 (52.0-197.0)	2.0	101.5 (57.8-190.5)	2.0	0.981
White meat	130.0 (52.0-260.0)	2.5	130.0 (52.0-227.5)	2.5	0.828
Meat and fish products (processed)	126.0 (48.3-203.4)	2.4	104.5 (54.5-306.5)	2.0	0.540
Fish and seafood	30.0 (6.8-73.6)	0.6	42.0 (27.0-57.0)	0.8	0.221
Milk and milk products	839.5 (538.8-1235.1)	16.1	769.5 (477.5-1168.3)	14.8	0.439
Eggs	131.3 (130.0-262.5)	2.5	132.5 (130.0-262.5)	2.6	0.265
Butter and other animal fats	260.0 (30.0-365.0)	5.0	52.0 (30.0-260.0)	1.0	0.180
Vegetable oils and fats	260.0 (130.0-365.0)	5	260.0 (130.0-365.0)	5	0.278
Sauces	32.5 (12.0-68.5)	0.6	60.0 (32.5-131.9)	1.2	0.026^a^
Coffee, tea, cocoa	1172.5 (732.5-1828.1)	22.6	1172.5 (553.1-1300.6)	22.6	0.256
Water	1825.0 (912.5-1825.0)	35.1	1825.0 (1140.6-1825.0)	35.1	0.206
Sweetened beverages	7.5 (0.0-50.9)	0.1	23.0 (5.0-84.5)	0.4	0.049^a^
Alcohol	28.3 (6.9-83.3)	0.5	51.8 (10.0-130.0)	1.0	0.301
Sweets, pastries	337.8 (122.8-556.6)	6.5	524.5 (350.3-734.8)	10.1	0.003^a^
Sugar, honey	53.3 (2.5-365.0)	1.0	145.0 (25.5-417.0)	2.8	0.065
Sweeteners and products containing them	32.5 (2.5-382.8)	0.6	15.8 (0.0-115.0)	0.3	0.049^a^
Fast food, snacks	74.5 (33.6-152.8)	1.4	111.0 (67.8-173.6)	2.1	0.091
Alternative products	0.0 (0.0-5.0)	-	3.8 (0.0-50.8)	0.1	0.014^a^

^a^P<0.05. Data are presented as the median (1st and 3rd quartiles). The groups were compared using Mann-Whitney U test. ‘Times/week’ is an approximate coefficient, assuming there are 52 weeks in a year.

**Table III:** Median 4-day nutrient intake by sex compared with the Latvian Ministry of Health recommended daily intake.

	Type 1 diabetes group (n=74)		Control group (n=32)		Daily average recommended amount (16,21)	
Nutrient	Male (n=28)	Female (n=46)	Male (n=13)	Female (n=19)	Male	Female
Energy, kcal	2,053.2 (1,822.1-2,863.6)	1,387.4^a^ (1,198.8-1,629.1)	2,096.6 (1,687.2-2,885.2)	1,789.7 (1,389.4-2,074.7)	2,290-2,960	1,840-2,360
Proteins	102.5 (78.0-129.0) g; 20 E%	63.6^b^ (49.7-77.1) g; 18 E%	98.9 (74.4-117.0) g; 19 E%	77.5 (55.1-99.0) g; 17 E%	10-20 E%	
Carbohydrates	203.9 (157.9-261.6) g; 40 E%	125.7 (93.1-172.5) g; 36 E%	216.0 (152.9-257.2) g; 41 E%	141.1 (108.9-213.7) g; 32 E%	45-60 E%	
Fats	95.1 (73.6-137.8) g; 42 E%	61.1^c^ (54.8-75.4) g; 40 E%	93.7 (68.5-133.6) g; 40 E%	80.7 (58.9-92.2) g; 41 E%	25-30 E%	
Fiber, g	23.9 (14.5-33.6)	14.0 (10.2-18.8)	16.9 (12.3-23.6)	17.0 (13.2-23.3)	25-35	

Data are presented as the median (1st and 3rd quartiles), as well as the average amount of macronutrients expressed as a percentage.

^a^P=0.003 vs. female participants in the control group,

^b^P=0.025 vs. female participants in the control group;

^c^P=0.003 vs. female participants in the control group. E%, percentage of total energy intake.

**Table IV:** Associations between food product consumption and markers of intestinal permeability and inflammation in the T1D group.

Outcome variable/predictor	OR (95% CI)	P-value
LPS/HDL ratio/Cereals and pasta	Model 1: 2.69 (1.05-6.90)	0.039^a^
	Model 2: 2.68 (1.04-6.87)	0.041^a^
	Model 3: 2.42 (0.92-6.35)	0.072
	Model 4: 2.47 (0.93-6.59)	0.071
	Model 5: 2.78 (1.01-7.66)	0.048^a^
LPS/HDL ratio/Red meat	Model 1: 2.71 (1.06-6.95)	0.038^a^
	Model 2: 2.63 (1.01-6.80)	0.047^a^
	Model 3: 2.30 (0.87-6.10)	0.094
	Model 4: 2.30 (0.87-6.10)	0.094
	Model 5: 2.27 (0.85-6.07)	0.103
LPS/Red meat	Model 1: 2.07 (1.17-8.02)	0.022^a^
	Model 2: 2.85 (1.08-7.56)	0.035^a^
	Model 3: 2.52 (0.93-6.81)	0.069
	Model 4: 2.52 (0.93-6.86)	0.071
	Model 5: 2.56 (0.94-7.02)	0.067
hs-CRP/Legumes	Model 1: 3.21 (1.14-9.04)	0.027^a^
	Model 2: 2.87 (0.99-8.29)	0.052
	Model 3: 3.01 (1.03-8.81)	0.045^a^
	Model 4: 3.31 (1.08-10.15)	0.037^a^
	Model 5: 4.91 (1.38-17.41)	0.014^a^
EndoCAb IgM/Sweeteners and products containing them	Model 1: 0.39 (0.15-1.00)	0.050
	Model 2: 0.38 (0.14-0.98)	0.046^a^
	Model 3: 0.37 (0.14-0.97)	0.044^a^
	Model 4: 0.36 (0.14-0.96)	0.042^a^
	Model 5: 0.36 (0.13-0.96)	0.040^a^
Calprotectin/Sweeteners and products containing them	Model 1: 0.36 (0.14-0.93)	0.036^a^
	Model 2: 0.35 (0.13-0.92)	0.033^a^
	Model 3: 0.32 (0.12-0.87)	0.025^a^
	Model 4: 0.32 (0.12-0.87)	0.025^a^
	Model 5: 0.32 (0.12-0.86)	0.025^a^
LPS/Water	Model 1: 2.50 (0.92-6.82)	0.073
	Model 2: 3.57 (1.16-10.99)	0.026^a^
	Model 3: 3.26 (1.06-10.05)	0.040^a^
	Model 4: 3.22 (1.04-9.96)	0.043^a^
	Model 5: 3.18 (1.01-10.06)	0.049^a^
hs-CRP/Coffee, tea and cocoa	Model 1: 3.99 (1.50-10.59)	0.006^a^
	Model 2: 3.86 (1.44-10.38)	0.007^a^
	Model 3: 3.99 (1.47-10.82)	0.007^a^
	Model 4: 3.73 (1.36-10.25)	0.011^a^
	Model 5: 3.95 (1.41-11.08)	0.009^a^

^a^P<0.05. Results of the binary logistic regression analysis with dichotomized inflammation/endotoxemia markers as the response variable in subjects with T1D. Food consumption was used as the predictor, categorized into two groups based on the median. Data are presented as OR with 95% CI and P-values. Model 1, univariate; Model 2, adjusted for BMI; Model 3, adjusted for BMI and diabetes duration; Model 4, adjusted for BMI, HbA1c and diabetes duration; Model 5, adjusted for BMI, HbA1c, diabetes duration and gastrointestinal symptom score. BMI, body mass index; EndoCAb, endogenous anti-endotoxin core antibody; HbA1c, glycated hemoglobin; HDL, high-density lipoprotein; hs-CRP, high-sensitivity C-reactive protein; LPS, lipopolysaccharide; T1D, type 1 diabetes.

**Table V:** Associations between macronutrient intake, hs-CRP and fecal calprotectin in the T1D group.

Outcome variable/predictor	OR (95% CI)	P-value
hs-CRP/Energy	Model 1: 0.29 (0.11-0.79)	0.015^a^
	Model 2: 0.30 (0.11-0.81)	0.018^a^
	Model 3: 0.28 (0.10-0.76)	0.013^a^
	Model 4: 0.25 (0.09-0.72)	0.011^a^
	Model 5: 0.26 (0.09-0.77)	0.015^a^
Calprotectin/Protein	Model 1: 3.00 (1.12-8.06)	0.029^a^
	Model 2: 3.01 (1.12-8.12)	0.030^a^
	Model 3: 2.86 (1.05-7.79)	0.040^a^
	Model 4: 2.86 (1.05-7.80)	0.040^a^
	Model 5: 3.37 (1.14-9.95)	0.028^a^
hs-CRP/Protein	Model 1: 0.31 (0.11-0.88)	0.027^a^
	Model 2: 0.32 (0.11-0.92)	0.034^a^
	Model 3: 0.29 (0.10-0.86)	0.025^a^
	Model 4: 0.26 (0.08-0.80)	0.018^a^
	Model 5: 0.27 (0.08-0.87)	0.028^a^
hs-CRP/Fat	Model 1: 0.31 (0.11-0.88)	0.027^a^
	Model 2: 0.32 (0.11-0.91)	0.032^a^
	Model 3: 0.28 (0.09-0.82)	0.021^a^
	Model 4: 0.28 (0.09-0.86)	0.026^a^
	Model 5: 0.29 (0.09-0.93)	0.037^a^
hs-CRP/Fiber	Model 1: 0.41 (0.15-1.12)	0.082
	Model 2: 0.38 (0.13-1.06)	0.063
	Model 3: 0.34 (0.12-0.99)	0.047^a^
	Model 4: 0.35 (0.12-1.04)	0.058
	Model 5: 0.35 (0.12-1.05)	0.061

^a^P<0.05. Results of the binary logistic regression analysis with hs-CRP and fecal calprotectin as response variables in T1D. Data are presented as OR with 95% CI and P-values. Model 1, univariate; Model 2, adjusted for BMI; Model 3, adjusted for BMI and diabetes duration; Model 4, adjusted for BMI, HbA1c and diabetes duration; Model 5, adjusted for BMI, HbA1c, diabetes duration and gastrointestinal symptom score. BMI, body mass index; HbA1c, glycated hemoglobin; hs-CRP, high-sensitivity C-reactive proteinT1D, type 1 diabetes.

## Data Availability

The data generated in the present study may be requested from the corresponding author.
