# Supplementary material for: Association of diet with intestinal permeability and inflammation markers in patients with type 1 diabetes
Source: Biomed Rep. 2026 Jul 29;25(4):108. doi: 10.3892/br.2026.2181 (PMC13454751; doi:10.3892/br.2026.2181)
Supplement: Supplementary file 1 [file Supplementary_Data1.pdf]

Table SI. Product groups.

Group number	Product group name	Products
1	Cereals, pasta	Breakfast cereals; grain porridge; buckwheat, cooked; couscous, cooked; quinoa, cooked; pasta, boiled; muesli; rice, cooked
2	Fast food, snacks	French fries, hot dogs, hamburgers (fast food restaurant), hamburgers, pizza, kebab, manufactured sandwich, instant soups, potato chips, corn chips, crackers, popcorn, dumplings
3	Sweets, pastries	Ice cream, pastries, cookies, wafers with filling, gingerbread, cream cakes, muffins, sheet cakes (apple, rhubarb, etc.), milk desserts, candy and sweets (chocolates, caramels, etc.), fruit jam, rye bread with additives (fruits, nuts, etc.)
4	Sweeteners and products containing them	Sugar substitutes, stevia powder, zero-calorie carbonated caffeinated drink (e.g. Coca-Cola Zero), sweets for diabetics, sugar-free cookies
5	Bread	Sweet and sour bread, rye bread, grain bread, white wheat bread, gluten-free white bread
6	Red meat	Pork, fatty, fresh, cooked; beef, cooked; lamb, cooked; game meat, cooked
7	White meat	Poultry meat, boiled, stewed
8	Meat and fish products (processed)	Sausages, canned meat, industrially smoked meat, sprats, canned fish, fish fingers, traditionally smoked dark fish (rural or wood-smoked)
9	Fish and seafood	Fish, cooked; seaweed; nori seaweed sheets
10	Eggs	Chicken eggs, fresh, cooked; quail eggs, boiled, fried
11	Milk and milk products	Milk 2%, fermented milk products, cream, sour cream products, cottage cheese 5%, cottage cheese desserts, cheese (gouda, Russian, etc.), cottage cheese 0.5%, low-fat cheese (up to 10% fat)
12	Alternative products	Plant milk (cow milk substitute), plant-based culinary cream (cream substitute), tofu, vegan cheese, soy, soy products
13	Sauces	Mayonnaise, soy sauce
14	Butter and other animal fats	Other animal fats Butter
15	Vegetable Oils and Fats	Vegetable oil, margarine
16	Fruits and berries	Fresh fruits, fresh berries, dried fruits
17	Vegetables (fresh, cooked)	Fresh vegetables; onions, fresh; garlic, fresh; vegetable soup (mixed vegetables); vegetable stew (various vegetables)
18	Pickled and salted vegetables	Mixed vegetables, pickled; vegetables, salted
19	Potatoes	Boiled potatoes, fried potatoes
20	Mushrooms (fresh and pickled)	Mushrooms, pickled mushrooms
21	Legumes	Legumes
22	Nuts, seeds	Nuts; peanuts, roasted, salted; seeds; peanut butter
23	Coffee, tea, cocoa	Coffee (brewed); instant coffee; coffee substitutes (chicory, barley, etc.), beverages; tea (beverage); cocoa, chocolate drink
24	Alcohol	Light beer, wine, alcoholic cocktails, strong alcoholic drinks
25	Sweetened beverages	Sweetened caffeinated drinks (e.g. Pepsi, cola); sweetened drinks (e.g. Fanta, Limpopo, etc.); kvass, malt drinks; energy drinks
26	Sugar, honey	Honey, sugar
27	Water	Water

Table SII. Medians for data grouping.

Variable	Median
Cereals, pasta, annual consumption coefficient	234.5
Fast food, snacks, annual consumption coefficient	87
Sweets, pastries, annual consumption coefficient	376
Sweeteners and products containing them, annual consumption coefficient	30
Bread, annual consumption coefficient	494
Red meat, annual consumption coefficient	109
White meat, annual consumption coefficient	130
Meat and fish products (processed), annual consumption coefficient	111
Fish and seafood, annual consumption coefficient	32.5
Eggs, annual consumption coefficient	132.5
Milk and milk products, annual consumption coefficient	834
Alternative products, annual consumption coefficient	0
Sauces, annual consumption coefficient	42
Butter and other animal fats, annual consumption coefficient	132.5
Vegetable oils and fats, annual consumption coefficient	260
Fruits and berries, annual consumption coefficient	377
Vegetables (fresh, cooked), annual consumption coefficient	689
Pickled and salted vegetables, annual consumption coefficient	24
Potatoes, annual consumption coefficient	104
Mushrooms (fresh and pickled), annual consumption coefficient	30
Legumes, annual consumption coefficient	30
Nuts, seeds, annual consumption coefficient	96.5
Coffee, tea, cocoa, annual consumption coefficient	1,172.5
Alcohol, annual consumption coefficient	32.5
Sweetened beverages, annual consumption coefficient	14.5
Sugar, honey, annual consumption coefficient	82
Water, annual consumption coefficient	1,825
LBP, µg/ml	11.059
LPS, EU/ml	0.366
LPS/HDL ratio	0.243
EndoCAb IgM, MMU/ml	45.979
EndoCAb IgG, GMU/ml	85.443
Calprotectin, µg/g	6.050
Energy (T1D), kcal	1,720.3
Protein (T1D), g	83.05
Carbohydrate (T1D), g	164.8
Fat (T1D), g	78.1
Fiber (T1D), g	18.95
Energy (Control), kcal	1,943.15
Protein (Control), g	88.2
Carbohydrate (Control), g	178.55
Fat (Control), g	87.2
Fiber (Control), g	16.95

EndoCAb, endogenous anti-endotoxin core antibody; HDL, high-density lipoprotein; LBP, LPS-binding protein; LPS, lipopolysaccharide; T1D, type 1 diabetes.

Table SIII. Levels of inflammatory and endotoxemia markers by frequency of food group consumption in T1D and control groups.

Food group	Variable	T1D group					Control group				
		Low consumption		Moderate-to-high consumption		P-value	Low consumption		Moderate-to-high consumption		P-value
		Median (Q1-Q3)	n	Median (Q1-Q3)	n		Median (Q1-Q3)	n	Median (Q1-Q3)	n	
Cereals, pasta	LBP	10.69 (7.75-15.53)	36	12.30 (8.0-14.38)	35	0.408	8.99 (6.23-14.73)	17	10.20 (8.68-14.19)	15	0.411
	LPS/HDL ratio	0.22 (0.17-0.26)	36	0.28 (0.22-0.34)	35	0.023[a]	0.25 (0.21-0.36)	17	0.22 (0.18-0.53)	14	0.544
	LPS	0.35 (0.30-0.44)	36	0.40 (0.31-0.48)	35	0.232	0.39 (0.33-0.51)	17	0.38 (0.30-0.53)	14	0.799
	EndoCAb IgM	45.96 (33.41-91.63)	35	42.97 (30.61-68.64)	35	0.507	56.31 (30.85-70.40)	17	41.35 (26.30-94.59)	15	0.455
	EndoCAb IgG	86.81 (66.68-132.67)	34	95.50 (54.88-161.07)	34	0.556	84.63 (42.38-148.32)	17	69.47 (58.03-87.71)	15	0.411
	hs-CRP	1.07 (0.49-2.07)	35	0.82 (0.36-1.75)	35	0.459	0.49 (0.27-0.90)	17	0.35 (0.24-0.76)	14	0.597
	Calprotectin	5.70 (2.70-13.30)	31	5.90 (2.30-19.10)	31	0.725	6.45 (3.93-8.95)	16	6.40 (3.68-12.08)	14	0.984
Bread	LBP	11.06 (7.93-17.80)	35	11.25 (7.80-13.67)	36	0.441	12.32 (6.41-14.18)	17	9.97 (7.20-15.10)	15	0.794
	LPS/HDL ratio	0.24 (0.17-0.28)	35	0.25 (0.19-0.34)	36	0.154	0.25 (0.21-0.44)	17	0.22 (0.19-0.42)	14	0.570
	LPS	0.35 (0.30-0.43)	35	0.40 (0.30-0.51)	36	0.206	0.36 (0.31-0.52)	17	0.39 (0.30-0.52)	14	0.922
	EndoCAb IgM	45.52 (33.92-91.90)	34	46.55 (29.91-64.55)	36	0.391	46.02 (28.16-64.87)	17	50.61 (26.21-94.59)	15	0.823
	EndoCAb IgG	104.03 (67.63-150.20)	35	87.71 (45.64-143.24)	33	0.222	80.71 (61.53-145.30)	17	70.24 (39.85-92.95)	15	0.350
	hs-CRP	0.75 (0.46-1.96)	34	0.98 (0.39-1.82)	36	0.948	0.29 (0.25-0.76)	17	0.52 (0.30-1.19)	14	0.262
	Calprotectin	4.00 (2.70-18.35)	29	6.20 (2.50-16.15)	33	0.949	6.10 (3.85-10.65)	16	6.80 (3.88-12.88)	14	0.697
Milk and milk products	LBP	12.22 (8.37-17.76)	35	10.89 (7.80-13.33)	36	0.151	10.81 (6.11-14.32)	18	10.08 (8.52-14.54)	14	0.442
	LPS/HDL ratio	0.24 (0.20-0.28)	35	0.25 (0.17-0.32)	36	0.687	0.30 (0.21-0.53)	17	0.22 (0.20-0.34)	14	0.279
	LPS	0.34 (0.30-0.45)	35	0.39 (0.31-0.48)	36	0.364	0.40 (0.30-0.59)	17	0.38 (0.31-0.46)	14	0.710
	EndoCAb IgM	41.11 (31.06-62.33)	34	56.18 (34.16-89.61)	36	0.184	33.38 (26.28-61.87)	18	56.54 (35.24-95.44)	14	0.168
	EndoCAb IgG	84.31 (56.74-140.74)	34	95.07 (67.50-150.91)	34	0.564	84.80 (67.54-117.58)	18	53.29 (31.51-85-41)	14	0.009[a]
	hs-CRP	1.00 (0.45-2.80)	34	0.84 (0.41-1.49)	36	0.350	0.52 (0.28-0.84)	18	0.30 (0.25-0.68)	13	0.332
	Calprotectin	8.10 (2.88-21.90)	30	4.65 (2.00-12.78)	32	0.101	7.05 (4.00-11.50)	16	5.25 (3.88-9.43)	14	0.525
Red meat	LBP	11.03 (7.86-	37	11.17 (7.86-	34	0.534	8.99 (6.41-	17	13.93 (7.20-	15	0.246

		16.31)		13.29)			13.87)		15.10)		
	LPS/HDL ratio	0.22 (0.17-0.28)	37	0.26 (0.20-0.37)	34	0.022[a]	0.22 (0.20-0.55)	17	0.34 (0.20-0.55)	14	0.109
	LPS	0.35 (0.30-0.40)	37	0.42 (0.31-0.57)	34	0.046[a]	0.35 (0.29-0.42)	17	0.46 (0.36-0.65)	14	0.029[a]
	EndoCAb IgM	46.56 (35.65-74.91)	36	45.0 (30.73-71.55)	34	0.438	50.68 (28.06-70.40)	17	37.77 (26.80-94.59)	15	0.710
	EndoCAb IgG	92.74 (46.23-149.12)	36	88.07 (69.36-141.28)	32	0.980	87.71 (54.71-170.66)	17	69.47 (45.79-84.63)	15	0.189
	hs-CRP	1.07 (0.40-2.50)	37	0.82 (0.44-1.39)	33	0.659	0.29 (0.25-0.68)	17	0.52 (0.30-1.43)	14	0.161
	Calprotectin	3.55 (2.70-15.65)	32	8.05 (3.05-18.13)	30	0.275	7.20 (4.00-11.60)	15	5.60 (3.80-9.00)	15	0.305
White meat	LBP	11.71 (7.77-16.20)	24	11.06 (7.91-13.55)	47	0.808	6.55 (5.92-8.10)	11	13.93 (9.41-15.11)	21	0.002[a]
	LPS/HDL ratio	0.24 (0.17-0.29)	24	0.25 (0.20-0.32)	47	0.296	0.22 (0.20-0.24)	11	0.34 (0.20-0.54)	20	0.025[a]
	LPS	0.35 (0.33-0.43)	24	0.37 (0.30-0.50)	47	0.679	0.35 (0.29-0.38)	11	0.45 (0.35-0.62)	20	0.009[a]
	EndoCAb IgM	52.25 (30.77-93.09)	23	45.37 (33.37-67.70)	47	0.392	59.53 (37.77-98.00)	11	32.34 (26.41-58.84)	21	0.056
	EndoCAb IgG	91.92 (60.10-142.30)	24	89.84 (67.25-144.89)	44	0.817	68.33 (44.91-181.65)	11	71.92 (53.29-96.88)	21	0.755
	hs-CRP	1.45 (0.60-2.52)	24	0.80 (0.34-1.42)	46	0.073	0.37 (0.24-0.77)	11	0.48 (0.25-0.75)	20	0.640
	Calprotectin	3.10 (0.70-9.10)	23	7.40 (3.0-18.90)	39	0.180	5.90 (3.90-7.20)	11	7.10 (3.80-11.40)	19	0.420
Fish and seafood	LBP	9.82 (7.55-15.61)	40	12.46 (9.12-13.69)	31	0.157	9.10 (6.45-14.19)	12	11.91 (7.41-14.87)	20	0.659
	LPS/HDL ratio	0.24 (0.20-0.35)	40	0.24 (0.17-0.29)	31	0.458	0.23 (0.19-0.48)	12	0.25 (0.20-0.37)	19	0.795
	LPS	0.35 (0.30-0.49)	40	0.37 (0.31-0.44)	31	0.808	0.37 (0.29-0.45)	12	0.40 (0.32-0.54)	19	0.435
	EndoCAb IgM	45.66 (30.77-69.63)	39	45.96 (34.31-76.66)	31	0.799	36.10 (27.48-65.73)	12	53.49 (26.86-88.92)	20	0.604
	EndoCAb IgG	82.70 (59.16-129.72)	39	97.99 (79.45-169.44)	29	0.066	72.20 (33.48-97.54)	12	71.08 (61.20-124.08)	20	0.408
	hs-CRP	0.78 (0.41-1.73)	40	1.08 (0.44-1.86)	30	0.780	0.48 (0.22-0.77)	12	0.41 (0.26-0.75)	19	0.857
	Calprotectin	5.90 (2.50-17.00)	33	5.70 (2.85-17.35)	29	0.994	6.30 (4.60-11.20)	11	6.50 (3.80-10.70)	19	0.672
Meat and fish products(processed)	LBP	10.70 (7.73-14.78)	34	11.67 (7.99-14.85)	37	0.427	10.08 (6.41-14.42)	18	10.92 (7.04-14.54)	14	0.985
	LPS/HDL ratio	0.24 (0.17-0.28)	34	0.25 (0.19-0.35)	37	0.629	0.26 (0.21-0.40)	18	0.24 (0.19-0.46)	13	0.984
	LPS	0.35 (0.31-0.43)	34	0.39 (0.29-0.50)	37	0.908	0.40 (0.31-0.53)	18	0.38 (0.30-0.51)	13	0.798

	EndoCAb IgM	59.59 (35.14-83.61)	33	41.66 (31.33-66.25)	37	0.140	48.35 (27.39-102.63)	18	44.19 (26.66-61.87)	14	0.442
	EndoCAb IgG	88.10 (69.76-143.24)	33	90.23 (49.51-149.45)	35	0.868	69.85 (60.16-108.34)	18	76.95 (31.51-111.91)	14	0.587
	hs-CRP	0.85 (0.38-2.55)	34	0.83 (0.49-1.66)	36	0.842	0.49 (0.26-0.91)	17	0.44 (0.25-0.64)	14	0.769
	Calprotectin	3.10 (1.9-8.5)	31	12.50 (3.30-19.20)	31	0.017[a]	6.10 (3.43-11.23)	16	7.05 (3.98-9.55)	14	0.448
Eggs	LBP	10.69 (7.77-13.77)	34	11.84 (8.23-16.01)	37	0.382	9.10 (6.28-14.02)	14	11.26 (8.10-15.11)	18	0.377
	LPS/HDL ratio	0.25 (0.19-0.29)	34	0.24 (0.17-0.33)	37	0.991	0.25 (0.19-0.44)	13	0.24 (0.20-0.42)	18	0.778
	LPS	0.36 (0.32-0.46)	34	0.37 (0.29-0.47)	37	0.756	0.36 (0.29-0.52)	13	0.42 (0.34-0.52)	18	0.312
	EndoCAb IgM	51.08 (32.44-81.73)	33	42.97 (32.43-66.33)	37	0.668	56.28 (26.53-97.25)	14	43.69 (27.43-63.61)	18	0.613
	EndoCAb IgG	88.78 (67.06-150.91)	34	92.60 (59.73-142.67)	34	0.893	70.13 (58.78-142.65)	14	76.11 (45.57-101.50)	18	0.955
	hs-CRP	0.70 (0.33-1.67)	33	1.13 (0.55-1.88)	37	0.167	0.54 (0.22-1.06)	14	0.37 (0.26-0.60)	17	0.799
	Calprotectin	4.65 (2.70-16.48)	32	6.80 (2.60-16.93)	30	0.709	6.65 (4.08-11.55)	12	6.20 (3.80-9.43)	18	0.692
Vegetables (fresh, cooked)	LBP	11.06 (7.75-16.21)	36	11.20 (8.0-13.57)	35	0.936	8.68 (6.41-14.00)	15	13.63 (7.77-14.73)	17	0.551
	LPS/HDL ratio	0.24 (0.18-0.33)	36	0.25 (0.18-0.29)	35	0.945	0.22 (0.18-0.52)	15	0.31 (0.21-0.37)	16	0.423
	LPS	0.34 (0.30-0.48)	36	0.37 (0.31-0.46)	35	0.364	0.34 (0.29-0.46)	15	0.44 (0.37-0.54)	16	0.049[a]
	EndoCAb IgM	45.37 (35.47-69.63)	35	47.13 (30.61-76.66)	35	0.609	50.61 (29.52-68.89)	15	46.02 (26.51-95.80)	17	1.000
	EndoCAb IgG	82.66 (48.95-127.50)	36	96.69 (73.65-149.98)	32	0.173	68.33 (44.91-100.81)	15	71.92 (59.45-117.24)	17	0.602
	hs-CRP	0.94 (0.33-2.37)	36	0.84 (0.49-1.60)	34	0.760	0.31 (0.22-0.62)	15	0.52 (0.27-1.07)	16	0.093
	Calprotectin	5.70 (2.70-22.70)	31	5.90 (2.70-16.10)	31	0.899	6.65 (4.08-10.60)	16	6.05 (3.30-10.93)	14	0.728
Pickled and salted vegetables	LBP	12.22 (8.34-15.36)	33	10.69 (7.77-14.27)	38	0.231	13.16 (8.83-14.92)	12	9.25 (6.23-14.12)	20	0.158
	LPS/HDL ratio	0.24 (0.19-0.28)	33	0.25 (0.17-0.32)	38	0.800	0.24 (0.21-0.52)	11	0.24 (0.20-0.37)	20	0.792
	LPS	0.35 (0.31-0.45)	33	0.37 (0.30-0.50)	38	0.661	0.44 (0.35-0.58)	11	0.38 (0.29-0.47)	20	0.298
	EndoCAb IgM	42.68 (31.97-68.45)	32	51.66 (33.27-80.41)	38	0.403	29.99 (26.31-55.97)	12	56.27 (31.58-88.92)	20	0.107
	EndoCAb IgG	88.78 (59.35-112.92)	32	94.11 (72.56-152.43)	36	0.308	66.96 (46.48-90.95)	12	81.34 (58.74-153.99)	20	0.366
	hs-CRP	0.85 (0.30-1.67)	32	0.83 (0.49-	38	0.458	0.28 (0.25-0.59)	11	0.52 (0.26-1.07)	20	0.212

				1.95)							
	Calprotectin	5.90 (2.30-30.90)	27	5.70 (3.0-15.80)	35	0.675	9.85 (5.40-12.08)	10	5.25 (3.43-8.25)	20	0.100
Potatoes	LBP	12.22 (7.92-16.13)	33	10.93 (7.66-13.48)	38	0.273	8.39 (6.41-14.28)	16	12.98 (9.35-14.87)	16	0.305
	LPS/HDL ratio	0.24 (0.20-0.31)	33	0.24 (0.17-0.30)	38	0.511	0.22 (0.19-0.31)	15	0.34 (0.20-0.54)	16	0.175
	LPS	0.37 (0.32-0.47)	33	0.35 (0.30-0.46)	38	0.549	0.34 (0.29-0.44)	15	0.46 (0.36-0.64)	16	0.024[a]
	EndoCAb IgM	45.21 (30.83-82.25)	32	46.39 (33.93-71.39)	38	0.637	53.76 (27.48-90.73)	16	41.90 (26.86-58.72)	16	0.423
	EndoCAb IgG	98.21 (57.43-147.79)	33	85.92 (60.63-146.00)	35	0.348	94.26 (52.34-176.15)	16	66.75 (48.85-83.97)	16	0.043[a]
	hs-CRP	0.69 (0.49-2.52)	32	0.98 (0.38-1.60)	38	0.976	0.39 (0.25-0.71)	16	0.49 (0.27-1.08)	15	0.379
	Calprotectin	4.80 (2.40-17.50)	28	6.05 (2.78-16.60)	34	0.645	6.30 (3.95-8.90)	13	6.50 (3.55-12.65)	17	0.934
Mushrooms (fresh, pickled)	LBP	10.86 (7.71-13.53)	32	11.67 (7.93-16.02)	39	0.272	8.10 (6.29-13.72)	13	13.73 (8.99-14.35)	19	0.117
	LPS/HDL ratio	0.26 (0.21-0.36)	32	0.24 (0.17-0.28)	39	0.058	0.22 (0.19-0.34)	13	0.28 (0.20-0.53)	18	0.567
	LPS	0.37 (0.30-0.54)	32	0.35 (0.31-0.44)	39	0.746	0.35 (0.29-0.46)	13	0.42 (0.33-0.57)	18	0.312
	EndoCAb IgM	45.37 (28.41-76.66)	31	47.13 (34.10-68.64)	39	0.763	50.68 (29.32-95.71)	13	46.02 (26.80-68.89)	19	0.705
	EndoCAb IgG	85.62 (46.23-141.28)	32	95.59 (73.50-148.59)	36	0.219	84.63 (45.35-115.87)	13	70.24 (60.87-103.55)	19	1.000
	hs-CRP	0.71 (0.35-1.49)	32	1.00 (0.56-2.49)	38	0.191	0.28 (0.23-0.52)	13	0.59 (0.29-1.18)	18	0.038[a]
	Calprotectin	3.35 (2.40-8.35)	24	8.00 (2.95-19.63)	38	0.117	7.10 (3.90-11.20)	11	5.60 (3.80-10.70)	19	0.641
Legumes	LBP	11.03 (7.36-13.41)	25	11.44 (7.98-16.08)	46	0.279	10.92 (6.24-14.07)	8	10.08 (6.93-14.92)	24	0.593
	LPS/HDL ratio	0.25 (0.19-0.31)	25	0.24 (0.17-0.31)	46	0.866	0.28 (0.19-0.50)	8	0.24 (0.20-0.35)	23	0.808
	LPS	0.34 (0.30-0.41)	25	0.38 (0.31-0.49)	46	0.248	0.36 (0.30-0.67)	8	0.40 (0.31-0.51)	23	0.947
	EndoCAb IgM	44.47 (30.97-69.33)	25	47.13 (33.76-78.55)	45	0.646	64.21 (38.31-119.58)	8	39.56 (26.66-59.84)	24	0.124
	EndoCAb IgG	80.62 (52.27-135.09)	25	95.16 (71.89-149.45)	43	0.220	76.48 (48.85-123.97)	8	71.08 (51.63-102.87)	24	0.848
	Hs-CRP	0.69 (0.28-1.26)	24	1.23 (0.56-2.17)	46	0.019[a]	0.51 (0.23-0.71)	8	0.41 (0.25-0.77)	23	0.912
	Calprotectin	6.85 (2.78-17.53)	22	4.60 (2.40-17.40)	40	0.596	7.05 (4.55-10.55)	8	5.75 (3.68-10.88)	22	0.420
Nuts, seeds	LBP	11.13 (7.87-13.30)	35	11.37 (7.81-16.21)	36	0.739	8.39 (6.45-13.88)	16	13.91 (9.06-15.38)	16	0.061

	LPS/HDL ratio	0.22 (0.17-0.27)	35	0.26 (0.21-0.35)	36	0.036[a]	0.24 (0.18-0.37)	15	0.28 (0.21-0.49)	16	0.545
	LPS	0.35 (0.30-0.42)	35	0.39 (0.30-0.52)	36	0.218	0.38 (0.29-0.58)	15	0.40 (0.32-0.50)	16	0.711
	EndoCAb IgM	46.39 (30.79-77.60)	34	45.67 (33.55-69.39)	36	0.760	36.10 (26.38-56.29)	16	58.18 (29.40-113.88)	16	0.080
	EndoCAb IgG	89.84 (59.53-137.78)	32	91.49 (65.80-159.08)	36	0.740	68.54 (41.12-97.54)	16	75.47 (61.57-124.08)	16	0.381
	hs-CRP	0.88 (0.45-1.86)	34	0.81 (0.41-1.82)	36	0.773	0.39 (0.24-0.61)	16	0.49 (0.27-1.05)	15	0.264
	Calprotectin	3.30 (2.50-18.35)	29	7.40 (3.05-16.15)	33	0.463	7.15 (4.15-10.65)	16	5.45 (3.68-10.93)	14	0.525
Fruits, berries	LBP	10.84 (7.56-14.17)	30	11.67 (7.96-15.19)	41	0.364	9.75 (7.65-14.05)	18	11.91 (6.35-15.19)	14	0.808
	LPS/HDL ratio	0.24 (0.19-0.29)	30	0.25 (0.17-0.32)	41	0.736	0.31 (0.20-0.54)	18	0.22 (0.20-0.32)	13	0.196
	LPS	0.34 (0.29-0.44)	30	0.40 (0.31-0.47)	41	0.119	0.41 (0.31-0.59)	18	0.39 (0.30-0.46)	13	0.489
	EndoCAb IgM	44.92 (30.38-64.53)	30	52.37 (34.16-76.75)	40	0.325	53.46 (29.48-62.86)	18	41.90 (25.89-103.96)	14	0.808
	EndoCAb IgG	82.70 (60.28-135.64)	29	95.16 (67.63-160.77)	39	0.272	75.09 (55.66-110.39)	18	71.08 (42.37-103.96)	14	0.694
	hs-CRP	0.81 (0.31-1.93)	29	0.86 (0.49-1.81)	41	0.877	0.32 (0.24-0.75)	18	0.54 (0.30-0.91)	13	0.170
	Calprotectin	8.60 (2.90-18.90)	27	3.90 (1.30-14.0)	35	0.101	6.75 (3.95-11.25)	18	5.95 (3.83-10.28)	12	0.723
Butter and other animal fats	LBP	10.27 (7.70-13.52)	32	12.22 (7.99-16.02)	39	0.155	9.64 (6.41-14.83)	20	11.91 (7.41-14.31)	12	0.623
	LPS/HDL ratio	0.25 (0.22-0.29)	32	0.23 (0.17-0.34)	39	0.361	0.28 (0.20-0.48)	20	0.24 (0.20-0.37)	11	0.640
	LPS	0.36 (0.31-0.47)	32	0.35 (0.30-0.46)	39	0.817	0.38 (0.31-0.52)	20	0.39 (0.29-0.51)	11	0.919
	EndoCAb IgM	53.66 (38.10-91.64)	31	41.34 (31.16-64.79)	39	0.129	53.49 (29.40-88.17)	20	41.90 (26.36-68.00)	12	0.431
	EndoCAb IgG	94.98 (78.39-153.18)	31	88.10 (59.54-130.65)	37	0.353	86.34 (64.06-152.48)	20	59.45 (30.28-79.47)	12	0.010[a]
	hs-CRP	0.80 (0.41-1.82)	32	1.11 (0.45-1.82)	38	0.728	0.48 (0.25-0.75)	20	0.41 (0.29-2.50)	11	0.476
	Calprotectin	5.70 (1.30-15.05)	25	5.90 (2.95-19.00)	37	0.397	6.65 (3.88-11.45)	18	6.05 (3.83-8.95)	12	0.755
Vegetable oils and fats	LBP	11.52 (7.90-16.41)	26	11.06 (7.82-13.63)	45	0.599	9.97 (7.23-14.88)	13	10.20 (6.55-14.06)	19	0.821
	LPS/HDL ratio	0.24 (0.20-0.30)	26	0.25 (0.17-0.31)	45	1.000	0.24 (0.21-0.33)	13	0.28 (0.20-0.53)	18	0.890
	LPS	0.35 (0.32-0.42)	26	0.37 (0.30-0.50)	45	0.676	0.35 (0.28-0.44)	13	0.42 (0.34-0.55)	18	0.146
	EndoCAb IgM	45.10 (33.48-	25	45.66 (32.43-	45	0.898	68.89 (30.62-	13	37.77 (26.61-	19	0.084

		77.72)		73.15)			108.25)		56.84)		
	EndoCAb IgG	84.31 (49.32-153.79)	26	95.07 (66.68-141.85)	42	0.762	63.69 (35.98-123.68)	13	80.71 (62.19-103.55)	19	0.254
	hs-CRP	0.97 (0.59-2.97)	26	0.84 (0.35-1.73)	44	0.316	0.49 (0.26-0.91)	13	0.44 (0.25-0.60)	18	0.514
	Calprotectin	4.65 (2.20-14.00)	22	6.05 (2.85-19.05)	40	0.422	6.3 (3.55-8.90)	13	7.0 (3.90-11.30)	17	0.621
Sauces	LBP	9.88 (7.66-13.56)	42	11.20 (9.43-20.06)	29	0.061	7.20 (6.07-13.82)	9	12.32 (8.68-15.10)	23	0.103
	LPS/HDL ratio	0.25 (0.20-0.33)	42	0.24 (0.16-0.28)	29	0.123	0.22 (0.18-0.26)	9	0.31 (0.20-0.54)	22	0.064
	LPS	0.37 (0.32-0.46)	42	0.35 (0.28-0.46)	29	0.272	0.31 (0.28-0.38)	9	0.44 (0.35-0.55)	22	0.026[a]
	EndoCAb IgM	45.37 (32.10-67.79)	41	53.66 (32.43-94.96)	29	0.180	46.02 (30.52-108.75)	9	50.61 (26.80-60.84)	23	0.321
	EndoCAb IgG	85.68 (49.35-127.50)	40	98.10 (72.56)	28	0.195	87.71 (61.53-159.31)	9	70.24 (45.79-92.95)	23	0.341
	hs-CRP	0.78 (0.30-1.84)	41	1.09 (0.63-1.82)	29	0.130	0.49 (0.27-0.70)	9	0.44 (0.25-0.83)	22	0.983
	Calprotectin	6.80 (2.70-16.88)	38	3.85 (2.40-17.38)	24	0.553	4.90 (3.43-10.28)	8	7.05 (3.98-10.83)	22	0.320
Fast food, snacks	LBP	11.21 (7.86-16.32)	40	11.03 (7.91-13.30)	31	0.324	13.73 (8.10-14.35)	11	9.52 (6.48-14.58)	21	0.434
	LPS/HDL ratio	0.24 (0.18-0.28)	40	0.24 (0.18-0.35)	31	0.570	0.22 (0.21-0.37)	11	0.28 (0.20-0.49)	20	0.670
	LPS	0.37 (0.31-0.49)	40	0.34 (0.30-0.42)	31	0.302	0.36 (0.34-0.48)	11	0.39 (0.29-0.54)	20	1.000
	EndoCAb IgM	53.66 (33.37-69.63)	39	42.97 (30.61-76.66)	31	0.353	41.36 (26.61-119.50)	11	50.68 (28.50-66.38)	21	0.938
	EndoCAb IgG	95.94 (65.51-155.71)	38	84.73 (60.46-107.79)	30	0.208	62.19 (45.79-92.95)	11	80.71 (56.86-133.95)	21	0.271
	hs-CRP	0.98 (0.49-2.71)	40	0.79 (0.35-1.43)	30	0.305	0.54 (0.27-0.75)	11	0.44 (0.24-0.97)	20	0.640
	Calprotectin	3.95 (2.70-17.40)	36	7.45 (2.60-16.80)	26	0.597	6.50 (3.90-9.00)	11	5.60 (3.80-11.20)	19	0.703
Sweets, pastries	LBP	12.93 (7.90-16.00)	41	10.28 (7.81-12.74)	30	0.279	11.85 (8.75-14.28)	10	9.86 (6.35-14.54)	22	0.562
	LPS/HDL ratio	0.25 (0.19-0.29)	41	0.24 (0.17-0.32)	30	0.617	0.27 (0.19-0.40)	10	0.24 (0.20-0.46)	21	0.950
	LPS	0.37 (0.33-0.50)	41	0.34 (0.28-0.42)	30	0.023[a]	0.39 (0.31-0.57)	10	0.39 (0.29-0.49)	21	0.917
	EndoCAb IgM	46.56 (34.11-68.93)	40	41.50 (31.41-79.45)	30	0.849	56.57 (28.67-77.58)	10	41.90 (26.68-69.88)	22	0.535
	EndoCAb IgG	97.00 (56.74-153.39)	38	87.91 (72.26-133.79)	30	0.621	81.21 (63.32-117.58)	10	71.08 (45.57-98.51)	22	0.411
	hs-CRP	0.75 (0.38-2.71)	40	0.98 (0.46-1.39)	30	0.510	0.60 (0.34-0.76)	10	0.31 (0.24-0.82)	21	0.233

	Calprotectin	6.05 (2.20-18.95)	38	5.40 (2.73-14.13)	24	0.745	7.45 (3.65-15.43)	10	6.20 (3.90-10.28)	20	0.779
Sweeteners and products containing them	LBP	11.13 (7.90-16.00)	29	11.37 (7.83-13.86)	42	0.925	9.97 (6.87-14.18)	17	10.20 (6.41-15.26)	15	0.970
	LPS/HDL ratio	0.24 (0.17-0.32)	29	0.24 (0.19-0.30)	42	0.726	0.22 (0.19-0.33)	17	0.32 (0.21-0.55)	14	0.077
	LPS	0.37 (0.31-0.52)	29	0.36 (0.29-0.45)	42	0.349	0.36 (0.30-0.44)	17	0.45 (0.31-0.60)	14	0.246
	EndoCAb IgM	60.48 (41.27-92-36)	29	38.60 (29.48-61.72)	41	0.008[a]	56.24 (33.56-83.25)	17	32.34 (26.21-60.84)	15	0.202
	EndoCAb IgG	109.51 (73.91-161.07)	26	86.82 (57.32-111.07)	42	0.086	65.16 (42.82-102.18)	17	81.98 (58.03-130.93)	15	0.295
	hs-CRP	1.18 (0.51-2.33)	28	0.80 (0.35-1.77)	42	0.436	0.49 (0.25-0.75)	17	0.44 (0.25-1.15)	14	0.984
	Calprotectin	8.05 (3.65-22.25)	28	3.2 (1.75-13.48)	34	0.040[a]	6.65 (3.88-10.28)	18	6.20 (3.85-11.08)	12	0.787
Coffee, tea, cocoa	LBP	10.64 (7.71-14.78)	33	12.07 (7.96-14.78)	38	0.447	13.63 (6.41-14.19)	15	9.52 (6.87-14.73)	17	0.911
	LPS/HDL ratio	0.24 (0.19-0.35)	33	0.24 (0.18-0.29)	38	0.580	0.23 (0.21-0.34)	14	0.25 (0.19-0.54)	17	0.984
	LPS	0.37 (0.32-0.48)	33	0.35 (0.30-0.43)	38	0.305	0.35 (0.30-0.44)	14	0.44 (0.33-0.56)	17	0.200
	EndoCAb IgM	52.95 (35.65-84.87)	32	44.92 (30.38-68.16)	38	0.383	56.31 (34.43-97.00)	15	32.34 (25.77-58.18)	17	0.049[a]
	EndoCAb IgG	88.10 (57.43-135.64)	33	94.97 (60.63-150.16)	35	0.663	69.47 (58.03-159.66)	15	81.98 (47.17-102.18)	17	0.911
	hs-CRP	0.70 (0.34-1.53)	33	1.28 (0.50-2.31)	37	0.074	0.49 (0.29-1.05)	15	0.37 (0.25-0.60)	16	0.202
	Calprotectin	3.70 (2.10-15.40)	32	6.80 (2.78-18.08)	30	0.334	5.90 (3.25-7.90)	13	7.10 (3.95-12.75)	17	0.079
Sweetened beverages	LBP	10.68 (7.56-16.29)	38	11.67 (8.34-13.56)	33	0.534	7.94 (6.23-13.91)	12	13.03 (8.32-15.22)	20	0.048[a]
	LPS/HDL ratio	0.25 (0.17-0.29)	38	0.24 (0.19-0.34)	33	0.991	0.21 (0.18-0.47)	12	0.31 (0.21-0.38)	19	0.177
	LPS	0.37 (0.34-0.49)	38	0.34 (0.29-0.43)	33	0.153	0.38 (0.29-0.55)	12	0.39 (0.34-0.51)	19	0.509
	EndoCAb IgM	47.13 (34.79-68.26)	37	42.97 (29.95-83.61)	33	0.800	43.69 (26.38-97.75)	12	53.43 (29.40-66.88)	20	0.863
	EndoCAb IgG	96.03 (66.24-153.60)	37	87.71 (59.93-112.93)	31	0.220	90.33 (58.74-170.48)	12	69.85 (45.13-84.88)	20	0.239
	hs-CRP	0.98 (0.44-2.49)	38	0.77 (0.41-1.33)	32	0.313	0.39 (0.24-0.61)	12	0.47 (0.27-0.77)	19	0.484
	Calprotectin	5.70 (0.90-15.90)	37	7.40 (3.05-17.65)	25	0.291	7.20 (3.80-11.60)	11	5.90 (3.90-8.80)	19	0.582
Alcohol	LBP	11.10 (7.80-13.64)	36	11.20 (7.91-16.35)	35	0.687	9.97 (8.06-14.19)	15	13.63 (6.36-15.18)	17	0.911
	LPS/HDL ratio	0.25 (0.21-0.31)	36	0.24 (0.17-	35	0.202	0.24 (0.21-0.52)	15	0.28 (0.20-0.37)	16	0.830

				0.30)						
	LPS	0.38 (0.31-0.49)	36	0.34 (0.30-0.42)	35	0.198	0.38 (0.29-0.58)	15	0.39 (0.32-0.47)	16	0.892
	EndoCAb IgM	53.66 (36.28-80.43)	35	42.97 (29.68-69.63)	35	0.140	41.35 (26.80-97.00)	15	50.61 (27.78-60.19)	17	0.710
	EndoCAb IgG	95.41 (59.73-142.89)	34	86.81 (64.18-146.87)	34	0.659	62.19 (44.91-84.96)	15	84.63 (66.75-148.32)	17	0.027[a]
	hs-CRP	0.82 (0.35-2.30)	36	0.95 (0.60-1.78)	34	0.814	0.32 (0.24-0.76)	14	0.49 (0.27-0.91)	17	0.421
	Calprotectin	4.50 (2.60-18.08)	30	8.05 (2.78-16.03)	32	0.612	8.80 (3.90-11.40)	15	4.90 (3.80-7.20)	15	0.250
Sugar, honey	LBP	11.76 (7.92-17.28)	40	11.06 (7.85-13.41)	31	0.385	12.98 (7.62-15.14)	14	9.75 (6.52-14.09)	18	0.613
	LPS/HDL ratio	0.24 (0.20-0.29)	40	0.24 (0.17-0.32)	31	0.719	0.31 (0.22-0.54)	14	0.22 (0.18-0.34)	17	0.077
	LPS	0.36 (0.31-0.48)	40	0.35 (0.30-0.43)	31	0.539	0.44 (0.34-0.55)	14	0.36 (0.28-0.47)	17	0.161
	EndoCAb IgM	45.37 (29.68-67.87)	39	60.03 (34.11-80.43)	31	0.279	33.38 (26.75-57.84)	14	56.28 (27.30-95.20)	18	0.301
	EndoCAb IgG	95.59 (57.85-144.46)	38	87.96 (64.00-146.87)	30	1.000	82.67 (57.79-157.46)	14	69.85 (44.30-94.92)	18	0.419
	hs-CRP	0.73 (0.49-1.79)	39	1.28 (0.36-1.84)	31	0.827	0.29 (0.25-0.51)	14	0.60 (0.29-1.06)	17	0.118
	Calprotectin	6.85 (2.20-18.08)	34	4.65 (2.93-12.78)	28	0.966	7.15 (4.93-11.35)	12	5.25 (3.68-9.28)	18	0.172
Water	LBP	11.13 (7.86-17.02)	25	11.13 (7.86-13.60)	46	0.665	9.44 (7.42-14.04)	8	11.15 (6.45-15.11)	24	0.782
	LPS/HDL ratio	0.24 (0.15-0.29)	25	0.25 (0.19-0.34)	46	0.233	0.24 (0.18-0.52)	7	0.24 (0.20-0.37)	24	0.945
	LPS	0.31 (0.29-0.43)	25	0.38 (0.33-0.49)	46	0.043[a]	0.44 (0.29-0.58)	7	0.38 (0.31-0.47)	24	0.764
	EndoCAb IgM	45.96 (30.23-73.15)	25	45.66 (33.39-74.73)	45	0.917	26.70 (26.24-32.73)	8	56.27 (33.70-88.92)	24	0.015[a]
	EndoCAb IgG	89.45 (62.03-157.39)	25	90.23 (60.63-129.72)	43	0.633	66.21 (45.82-83.76)	8	81.34 (58.74-124.08)	24	0.357
	hs-CRP	1.16 (0.64-2.03)	24	0.73 (0.38-1.75)	46	0.198	0.26 (0.21-0.41)	7	0.52 (0.27-0.98)	24	0.033[a]
	Calprotectin	6.30 (2.75-18.50)	25	3.90 (2.50-15.95)	37	0.656	9.0 (3.90-11.40)	7	6.30 (3.80-8.80)	23	0.564

[a]P<0.05. Food product groups are divided into two groups (‘low’ and ‘moderate-to-high’ consumption) according to the median (Table SII), except for hs-CRP, which was categorised using the clinically established threshold of low-grade inflammation, 1 mg/l (1). Differences between groups were determined by the Mann-Whitney U test. EndoCAb, endogenous anti-endotoxin core antibody; HDL, high-density lipoprotein; hs-CRP, high-sensitivity C-reactive protein; LBP, LPS-binding protein; LPS, lipopolysaccharide; T1D, type 1 diabetes.

Table SIV. Associations between food group consumption and markers of intestinal permeability and inflammation in the control group.

Outcome variable/predictor	OR (95% CI)	P-value
EndoCAb IgG/Butter and other animal fats	Model 1: 0.08 (0.01; 0.72)	0.024[a]
	Model 2: 0.09 (0.01; 0.78)	0.029[a]
	Model 3: 0.07 (0.01; 0.66)	0.021[a]
	Model 4: 0.08 (0.08; 0.82)	0.033[a]
LPS/Sauces	Model 1: 5.63 (1.02; 31.10)	0.048[a]
	Model 2: 5.66 (1.02; 31.45)	0.048[a]
	Model 3: 5.73 (1.02; 32.16)	0.047[a]
	Model 4: 4.50 (0.64; 31.60)	0.130
EndoCAb IgM/Coffee, tea and cocoa	Model 1: 0.22 (0.06; 0.86)	0.030[a]
	Model 2: 0.23 (0.05; 1.00)	0.050[a]
	Model 3: 0.20 (0.04; 0.94)	0.042[a]
	Model 4: 0.08 (0.01; 0.76)	0.027[a]
EndoCAb IgM/Nuts and seeds	Model 1: 3.67 (0.95; 14.09)	0.059
	Model 2: 7.15 (1.35; 37.91)	0.021[a]
	Model 3: 6.65 (1.25; 35.37)	0.026[a]
	Model 4: 6.04 (0.92; 39.59)	0.061
EndoCAb IgG/Potatoes	Model 1: 0.11 (0.02; 0.63)	0.013[a]
	Model 2: 0.11 (0.02; 0.64)	0.014[a]
	Model 3: 0.10 (0.02; 0.61)	0.013[a]
	Model 4: 0.11 (0.02; 0.69)	0.018[a]
LPS/Vegetables (fresh, cooked)	Model 1: 3.90 (1.00; 15.28)	0.051
	Model 2: 3.90 (0.99; 15.27)	0.051
	Model 3: 4.16 (1.02; 17.02)	0.048[a]
	Model 4: 10.19 (1.29; 80.47)	0.028[a]

[a]P<0.05. Results of the binary logistic regression analysis with the presence of inflammation/endotoxemia markers as the response variable in the control group. Data are presented as OR with 95% CI and P-values. Model 1, univariate; Model 2, adjusted for BMI; Model 3, adjusted for BMI and HbA1c; Model 4, adjusted for BMI, HbA1c and gastrointestinal symptom score. BMI, body mass index; EndoCAb, endogenous anti-endotoxin core antibody; HbA1c, glycated hemoglobin; LPS, lipopolysaccharide.

Table SV. Associations between macronutrient intake and markers of endotoxemia and inflammation in the control group.

Outcome variable/predictor	OR (95% CI)	P-value
EndoCAb IgM/Energy	Model 1: 0.23 (0.06; 0.95)	0.042[a]
	Model 2: 0.29 (0.06; 1.30)	0.105
	Model 3: 0.32 (0.06; 1.71)	0.181
	Model 4: 0.22 (0.03; 1.58)	0.133
EndoCAb IgM/Carbohydrate	Model 1: 0.26 (0.07; 1.00)	0.051
	Model 2: 0.18 (0.04; 0.88)	0.034[a]
	Model 3: 0.19 (0.04; 1.04)	0.055
	Model 4: 0.08 (0.01; 0.75)	0.027[a]
LPS/Fat	Model 1: 4.57 (1.14; 18.41)	0.033[a]
	Model 2: 4.58 (1.14; 18.48)	0.032[a]
	Model 3: 5.67 (1.22; 26.43)	0.027[a]
	Model 4: 2.41 (0.42; 13.66)	0.322
Calprotectin/Fat	Model 1: 3.43 (0.88; 13.39)	0.076
	Model 2: 3.52 (0.88; 14.11)	0.076
	Model 3: 6.00 (1.11; 32.31)	0.037[a]
	Model 4: 4.14 (0.70; 24.67)	0.118
LPS/Fiber	Model 1: 4.58 (1.16; 18.10)	0.030[a]
	Model 2: 4.80 (1.18; 19.44)	0.028[a]
	Model 3: 5.08 (1.21; 21.39)	0.027[a]
	Model 4: 8.12 (0.90; 72.90)	0.062
hs-CRP/Fiber	Model 1: 8.33 (0.87; 80.10)	0.066
	Model 2: 13.18 (1.09; 160.08)	0.043[a]
	Model 3: 15.67 (1.27; 193.74)	0.032[a]
	Model 4: 33.56 (1.12; 1009.28)	0.043[a]

[a]P<0.05. Results of the binary logistic regression analysis with the presence of inflammation/endotoxemia markers as the response variable in the control group. Data are presented as OR with 95% CI and P-values. Model 1, univariate; Model 2, adjusted for BMI; Model 3, adjusted for BMI and HbA1c; Model 4, adjusted for BMI, HbA1c and gastrointestinal symptom score. BMI, body mass index; EndoCAb, endogenous anti-endotoxin core antibody; HbA1c, glycated hemoglobin; hs-CRP, high-sensitivity C-reactive protein; LPS, lipopolysaccharide.

Reference

1. Pearson TA, Mensah GA, Alexander RW, *et al*: Markers of inflammation and cardiovascular disease. Circulation 107: 499-511, 2003.
